# A108 ASSESSING THE RELATIONSHIP BETWEEN MENTAL HEALTH, QUALITY OF LIFE, AND ACCESSING CARE IN PREGNANT IBD PATIENTS

**DOI:** 10.1093/jcag/gwae059.108

**Published:** 2025-02-10

**Authors:** V Srikanth, V Premjeyanth, S Perera, K O’Connor, N Krugliak, V Huang

**Affiliations:** Gastroenterology and Hepatology, Sinai Health, Toronto, ON, Canada; Gastroenterology and Hepatology, Sinai Health, Toronto, ON, Canada; Gastroenterology and Hepatology, Sinai Health, Toronto, ON, Canada; Gastroenterology and Hepatology, Sinai Health, Toronto, ON, Canada; Gastroenterology and Hepatology, Sinai Health, Toronto, ON, Canada; Gastroenterology and Hepatology, Sinai Health, Toronto, ON, Canada

## Abstract

**Background:**

Individuals with Inflammatory Bowel Disease (IBD) are at an increased risk of depression and anxiety, which are correlated with IBD disease activity and reduced quality of life. However, the impact of mental health on pregnant persons with IBD and the barriers to mental health care remain less clear.

**Aims:**

To explore associations between mental health, quality of life, and barriers to addressing mental health concerns in pregnant persons with IBD.

**Methods:**

We conducted an anonymous, cross-sectional survey of pregnant IBD patients. Data collected included demographics, IBD disease activity via modified Harvey Bradshaw Index (mHBI) for Crohn’s disease (CD) and the Partial Mayo Score 6 (pMayo6) for ulcerative colitis (UC), mental health scores (PHQ-9 and GAD-7), and IBD-related quality of life using the Short Inflammatory Bowel Disease Questionnaire (SIBDQ). We also explored patient perceptions of mental health stigma, barriers to accessing care, and comfort in discussing these concerns. Chi-square analysis and Fisher’s Exact Test were employed for categorical comparisons, and the Mann-Whitney U test was used to compare mental health scores with SIBDQ.

**Results:**

Fifty-seven surveys were analyzed (52.6% CD, 47.4% UC). The mean age was 33.98 years (SD 3.734), and 22.7% had active disease (mHBI ≥ 5 or pMayo6 > 1). Elevated mental health scores (PHQ-9 and/or GAD-7 ≥ 5) were observed in 57.9% of participants, with 7% having high PHQ-9 scores, 12.3% having high GAD-7 scores, and 38.6% having both. The median SIBDQ score was 57 (IQR 12). A significant association was observed between lower SIBDQ scores and the presence of both an elevated PHQ-9 and GAD-7 score (p < 0.001), while neither score alone had a significant effect.

17.5% reported feeling stigmatized when discussing mental health, highlighting concerns of being perceived as weak, some in their role as maternal figures. A statistically significant association was observed between a clinical diagnosis of both depression and anxiety and feeling stigmatized. 12.3% reported cultural deterrents, noting language, negative cultural connotations, and lack of understanding within their communities. Ethnicity, education, and marital status did not significantly affect satisfaction with available supports, experiencing stigma or barriers to access. There was no significant association between Caucasian versus non-Caucasian patients’ preference for discussing these concerns with a physician (GP, IBD specialist, gastroenterologist, OBGYN, psychiatrist) or family/friends.

**Conclusions:**

Pregnant IBD patients with both elevated PHQ-9 and GAD-7 scores may experience diminished quality of life, underscoring the need to address mental health concerns in disease management. Further research is needed to explore the influence of these factors on maternal-neonatal outcomes.

**Funding Agencies:**

University of Toronto: UT GE-HEP NEW INVESTIGATOR PILOT AWARD

